# Supplementary material for: FtsZ phosphorylation modulates tail-core binding to tune cell division in Bacillus subtilis
Source: PLoS One. 2025 Dec 29;20(12):e0337820. doi: 10.1371/journal.pone.0337820 (PMC12747378; doi:10.1371/journal.pone.0337820)
Supplement: S1 Raw Image — (PDF) [file pone.0337820.s001.pdf]

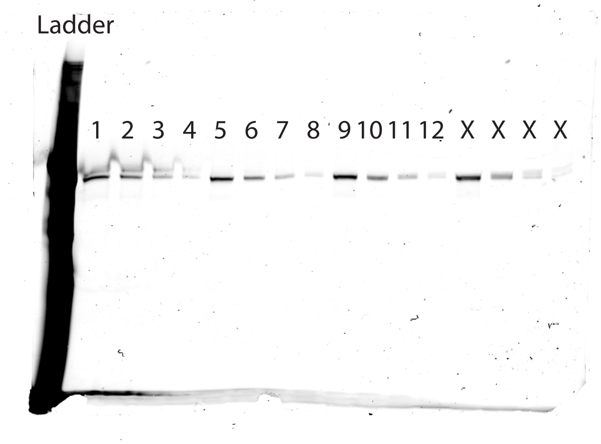

Related to Figure S2A

PhosTag gel electrophoresis
FtsZ-HaloTag conjugated to TMR dye

Each group is a 2-fold serial dilutions of cell lysate:
1-4: FtsZ WT
5-8: FtsZ S333A
9-12: FtsZ WT in a ΔprkC background

Imaged on an Azure Sapphire, 520 nm laser line

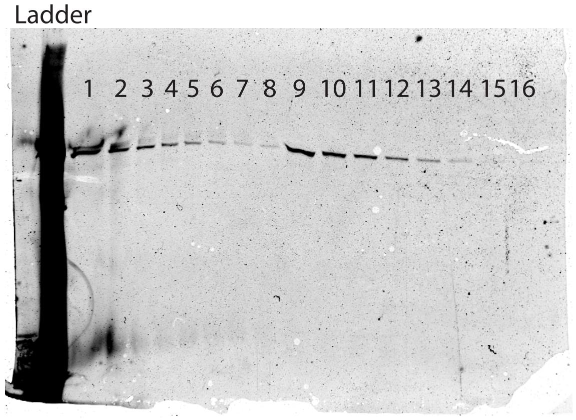

Related to Figure S2B

PhosTag gel electrophoresis
FtsZ-HaloTag conjugated to TMR dye

Each group is a 2-fold serial dilutions of cell lysate:
1-8: FtsZ WT
9-16: FtsZ S329A-S333A double-mutant

Imaged on an Azure Sapphire, 520 nm laser line

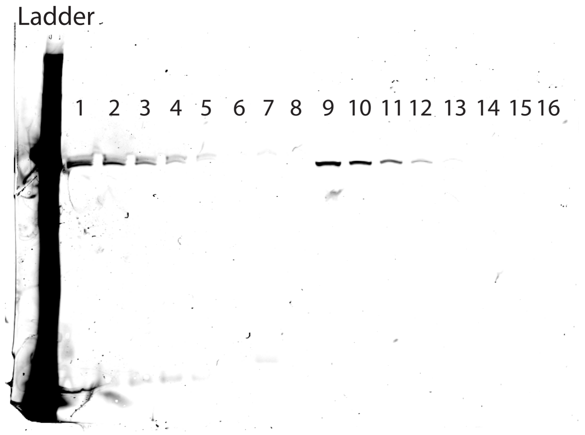

Related to Figure S2C

PhosTag gel electrophoresis
FtsZ-HaloTag conjugated to TMR dye

Each group is a 2-fold serial dilutions of cell lysate:
1-8: FtsZ S329A
9-16: FtsZ S333A

Imaged on an Azure Sapphire, 520 nm laser line

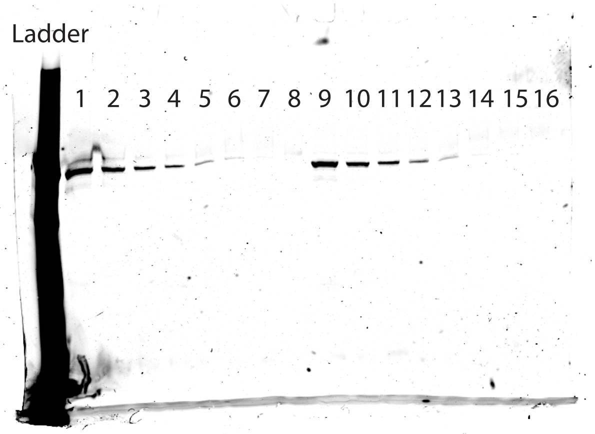

Related to Figure S2D

PhosTag gel electrophoresis
FtsZ-HaloTag conjugated to TMR dye

Each group is a 2-fold serial dilutions of cell lysate:
1-8: FtsZ WT
9-16: FtsZ WT in a ΔprkC background

Imaged on an Azure Sapphire, 520 nm laser line

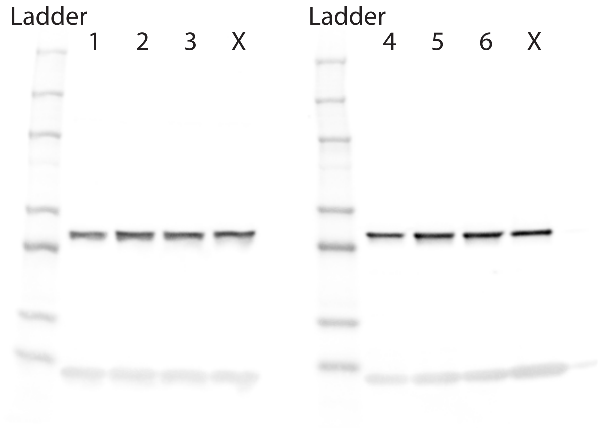

Related to Figure S4D (top)

Immunoblot of FtsZ in whole cell lysate
Rabbit polyclonal anti-FtsZ primary antibody
HRP-conjugated goat anti-rabbit secondary antibody

1. FtsZ WT - S7 minimal medium
2. FtsZ S333A - S7 minimal medium
3. FtsZ S333E - S7 minimal medium
4. FtsZ WT - LB rich medium
5. FtsZ S333A - LB rich medium
6. FtsZ S333E - LB rich medium

Imaged on BioRad GelDoc

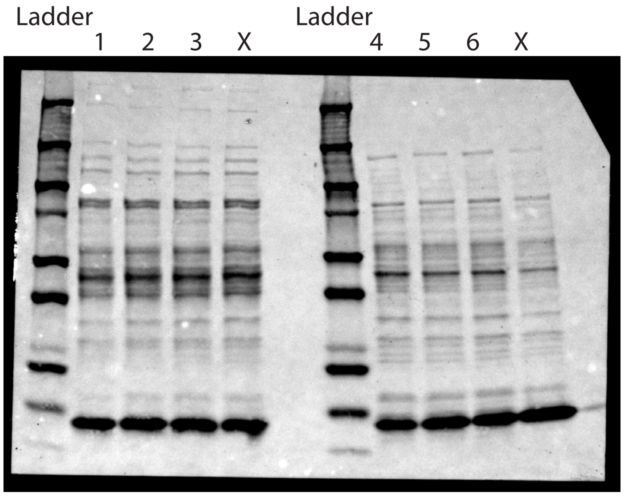

Related to Figure S4D (bottom)

Ponceau stain of the same blot as above

1. FtsZ WT - S7 minimal medium
2. FtsZ S333A - S7 minimal medium
3. FtsZ S333E - S7 minimal medium
4. FtsZ WT - LB rich medium
5. FtsZ S333A - LB rich medium
6. FtsZ S333E - LB rich medium

Imaged on BioRad GelDoc
