# Supplementary material for: FtsZ phosphorylation modulates tail-core binding to tune cell division in Bacillus subtilis
Source: PLoS One. 2025 Dec 29;20(12):e0337820. doi: 10.1371/journal.pone.0337820 (PMC12747378; doi:10.1371/journal.pone.0337820)
Supplement: S1 Appendix — Contains Tables A-F, detailed construction protocols, sequences, and supplementary references. (DOCX) [file pone.0337820.s015.docx]

Supplementary Information for

FtsZ phosphorylation modulates tail-core binding

to tune cell division in *Bacillus subtilis*

William J. Mallard^1†^, Vincent V. Pham^1†^

^1^Department of Molecular and Cellular Biology, Harvard University, Cambridge, Massachusetts 02138, USA

^†^These authors are joint corresponding authors.

* Email: wmallard@fas.harvard.edu (WM); vpham@g.harvard.edu (VP)

**Supplementary Tables**

**Table A. FtsZ CTL phosphosites across species**

| **Species** | **FtsZ Core**  **Phosphosite** | **FtsZ Tail**  **Phosphosite** | **Kinase** | **References** |
| --- | --- | --- | --- | --- |
| Bacillus subtilis | — | S329, S333 | PrkC | [1–3] |
| Corynebacterium glutamicum | T63 | S353, T388 | PknL | [4] |
| Deinococcus radiodurans | S235 | S335 | RqkA | [5] |
| Mycobacterium tuberculosis | — | — | PknA | [6] |
| Staphylococcus aureus | T128, T133, T145 | T365 | PknB | [7] |
| Streptococcus agalactiae | T7 | T357, S359 | Stk1 | [8] |
| Streptococcus pneumoniae | — | — | StkP | [9] |
| Streptomyces coelicolor | — | S319, S387 | PkaE | [10–13] |

**Table B. Secondary structure predictions in the FtsZ C-terminal linker**

| **Software** | **Region Size** | **Sequence** | **Prediction** | **Reference** |
| --- | --- | --- | --- | --- |
| GOR | 7aa | NQSIKTH | Beta strand | [14] |
| SOPMA | 6aa | LNQSIK | Alpha helix | [15] |
| PSIPRED | 8aa | LNQSIKTH | Alpha helix | [16] |
| YASPIN | 8aa | LNQSIKTH | Alpha helix | [17] |
| LS2P | 8aa | LNQSIKTH | Mixed alpha and  beta propensity | [18] |
| DISOPRED3 | 21aa | Q326 – P346 | Ordered region | [19] |
| AlphaFold3 | 8aa | LNQSIKTH | Alpha helix | [20] |

**Table C. *B. subtilis* strains used in this study**

| **Description** | **Strain** | **Genotype** | **Source** |
| --- | --- | --- | --- |
| WT | Py79 | *prototroph* | [21] |
|  |  |  |  |
| FtsZ(WT) | bWM200 | *amyE::erm-Phyperspank-ftsA-ftsZ*  *ftsAZ::spec* | this study |
| FtsZ(S333A) | bWM236 | *amyE::erm-Phyperspank-ftsA-ftsZ(S333A)*  *ftsAZ::spec* | this study |
| FtsZ(S333E) | bWM237 | *amyE::erm-Phyperspank-ftsA-ftsZ(S333E)*  *ftsAZ::spec* | this study |
|  |  |  |  |
| Halo-FtsZ(WT) | bAB309 | *amyE::erm-Phyperspank-ftsA-HaloTag-15aa-ftsZ* | this study |
| Halo-FtsZ(S329A) | bWM8 | *amyE::erm-Phyperspank-ftsA-HaloTag-15aa-ftsZ(S329A)* | this study |
| Halo-FtsZ(S333A) | bWM9 | *amyE::erm-Phyperspank-ftsA-HaloTag-15aa-ftsZ(S333A)* | this study |
| Halo-FtsZ (S329A,S333A) | bWM10 | *amyE::erm-Phyperspank-ftsA-HaloTag-15aa-ftsZ(S329A,S333A)* | this study |
| Halo-FtsZ ∆prkC | bWM106 | *amyE::erm-Phyperspank-ftsA-HaloTag-15aa-ftsZ*  *prkC::scar‡* | this study |

‡ ”scar” is a *lox72* site (34bp) produced by Cre-mediated excision of *lox71/lox66* [22].

**Table D. Intermediate strains used in this study**

| **Description** | **Strain** | **Genotype** | **Source** |
| --- | --- | --- | --- |
| P*hyperspank*-FtsZ(WT) | bWM98 | *amyE::erm-Phyperspank-ftsA-ftsZ* | this study |
| FtsZ(WT) | bWM174 | *amyE::erm-Paz-ftsA-ftsZ* | this study |
| FtsZ(S333A) | bWM233 | *amyE::erm-Paz-ftsA-ftsZ(S333A)* | this study |
| FtsZ(S333E) | bWM234 | *amyE::erm-Paz-ftsA-ftsZ(S333E)* | this study |
| ∆PrkC | bYS542 | *prkC::scar‡* | [23] |

**Table E. Plasmids used in this study**

| **Description** | **Plasmid** | **Genotype** | **Source** |
| --- | --- | --- | --- |
| FtsZ | pWM1 | pET28a::6xHis-TEV-FtsZ | this study |
| FtsZ_Core | pWM2 | pET28a::6xHis-TEV-FtsZ_Core | this study |
| FtsZ_Tail | pWM3 | pGEX-6P-3::GST-3C-FtsZ_Tail | this study |
| MinC_N_ | pWM4 | pGEX-6P-1::GST-3C-MinC_N_ | this study |

**Table F. Oligonucleotides used in this study**

| **Oligo Name** | **Sequence (5' to 3')** |
| --- | --- |
| oAB13 | CCAGTACCGATTTCTGCCATGCTAAATCCTCCTAATCTGCCGAATG |
| oAB14 | TGGCCTGAGCCCGGTCCCTGGCCAGATCCCTCGAGGCCGCTGATTTCTAAGGTAGAAAG |
| oAB76 | GAACGGTACTGAGCGAGGGAGCAGAAGTATTTGTTTCCGGTTTCT |
| oAB78 | AGCGGATAACAATTAAGCTTTAAGGAGGAACTACCATGAACAACAATGAACTTTACGTC |
| oAB94 | CTTTCGGTAAGTCCCGTCTAGCCTTGCCCTTAGCCGCGTTTATTACGGTTTC |
| oAB140 | GGACCGGGCTCAGGCCAAGGAAGCGGCATGTTGGAGTTCGAAACAAACATAGACG |
| oDS487 | CAGGGAGCACTGGTCAACTACCG |
| oDS488 | TTCTGCTCCCTCGCTCAGTACCG |
| oJE32 | ATGGCAGAAATCGGTACTGG |
| oMD108 | ACGAACGGTAGTTGACCAGTGCTCCCTGTCTTGACACTCCTTATTTGATTTTTTGAAGAC |
| oMD191 | TTTGGATGGATTCAGCCCGATTG |
| oMD196 | GGGCAAGGCTAGACGGG |
| oMD197 | TCACATACTCGTTTCCAAACGGATC |
| oMD232 | GGTAGTTCCTCCTTAAAGCTTAATTGTTATCCGCTCACAAT |
| oMD234 | ATACGAACGGTACTGAGCGAGGGAGCAGAATAATGGATTTCCTTACGCGAAATACG |
| oWM1 | ACGTGACGAAGCCTCAGCGTCCAAGCTTAAATCAAGCAATCAAAACACACAATCAAAGTG |
| oWM2 | CTCACGCTTCGGAACACTTTGATTGTGTGTTTTGATTGCTTGATTTAAGCTTGGACGCTG |
| oWM3 | ACGTGACGAAGCCTCAGCGTCCAAGCTTAAATCAAGAGATCAAAACACACAATCAAAGTG |
| oWM4 | CTCACGCTTCGGAACACTTTGATTGTGTGTTTTGATCTCTTGATTTAAGCTTGGACGCTG |
| oWM70 | CAAGAGAAGGACGTGACGAAGCCTCAGCGTCCAGCATTAAATCAAAGCATCAAAACACAC |
| oWM71 | TTCGGAACACTTTGATTGTGTGTTTTGATGCTTTGATTTAATGCTGGACGCTGAGGCTTC |
| oWM112 | CTATTTCAATGGCAGTTACGAAATTACACC |
| oWM113 | CCCTTGAATTAGTAAAGAGGTGTAATTTCG |
| oWM203 | TTTGATTGCTTGATTTAATGCTGGACGCTGAGGCTTCG |
| oWM204 | CCTCAGCGTCCAGCATTAAATCAAGCAATCAAAACACACAATCAAAGTGTTCC |
| oWM414 | TCAGCTGTCAATGGATCATATTTCTGTGTCGGCAG |
| oWM415 | ACGCAACAACCGTGCCAGTTCCATC |
| oWM416 | CGAACGGTAGTTGACCAGTGCTCCCTGTCTATGGCACCTCCTCACATTTCG |
| oWM417 | CGAACGGTACTGAGCGAGGGAGCAGAATGTAAAGGACAAAATCGTTTTCG |
| oWM612 | GATCTGGAAGTTCTGTTCCAGGGGCCCATGAAGACCAAAAAGCAGCAATATG |
| oWM613 | CGAGGCAGATCGTCAGTCAGTCACGATTTAAGTAATGACTTCACTGTCAATAGAATGA |

**Supplementary Methods**

**Strain Construction**

**bWM98** [*amyE::erm-Phyperspank-ftsA-ftsZ*] was generated by transforming PY79 with a Gibson assembly consisting of three fragments:

1. **amyE(up)-erm(5’)**: Created by overlap extension PCR of:
   - PCR with primers oMD191 and oMD108 using PY79 genomic DNA as template.
   - PCR with primers oDS487 and oWM113 using plasmid pWX467 as template.
2. **erm(3’)-Phyperspank**: Created by overlap extension PCR of:
   - PCR with primers oWM112 and oDS488 using plasmid pWX467 as template.
   - PCR with primers oMD234 and oMD232 using plasmid pDR111 as template.
3. **ftsAZ-amyE(down)**: Created by overlap extension PCR of:
   - PCR with primers oAB78 and oAB94 using PY79 genomic DNA as template.
   - PCR with primers oMD196 and oMD197 using PY79 genomic DNA as template.

The genotype was confirmed via PCR and Sanger sequencing of the entire *amyE* locus with 2X coverage.

**bWM174** [*amyE::erm-Paz-ftsA-ftsZ*] was generated by transforming PY79 with a Gibson assembly consisting of three fragments:

1. **amyE(up)-erm(5’)**: PCR with primers oMD191 and oWM113 using bWM98 genomic DNA as template.
2. **erm(3’)-Paz-ftsAZ**: Created by overlap extension PCR of:
   - PCR with primers oWM112 and oDS488 using plasmid pWX467 as template.
   - PCR with primers oAB76 and oAB94 using PY79 genomic DNA as template.
3. **amyE(down)**: PCR with primers oMD196 and oMD197 using PY79 genomic DNA as template.

The genotype was confirmed via PCR and Nanopore sequencing.

**bWM233** [*amyE::erm-Paz-ftsA-ftsZ(S333A)*] was generated by transforming PY79 with a Gibson assembly consisting of three fragments:

1. **amyE(up)-erm(5’)**: PCR with primers oMD191 and oWM113 using bWM98 genomic DNA as template.
2. **erm(3’)-Paz-ftsA-ftsZ(S333A)**: PCR with primers oWM112 and oWM2 using bWM174 genomic DNA as template.
3. **ftsZ(S333A)-amyE(down)**: PCR with primers oWM1 and oMD197 using bWM174 genomic DNA as template.

The genotype was confirmed via PCR and Nanopore sequencing.

**bWM234** [*amyE::erm-Paz-ftsA-ftsZ(S333E)*] was generated by transforming PY79 with a Gibson assembly consisting of three fragments:

1. **amyE(up)-erm(5’)**: PCR with primers oMD191 and oWM113 using bWM98 genomic DNA as template.
2. **erm(3’)-Paz-ftsA-ftsZ(S333E)**: PCR with primers oWM112 and oWM4 using bWM174 genomic DNA as template.
3. **ftsZ(S333E)-amyE(down)**: PCR with primers oWM3 and oMD197 using bWM174 genomic DNA as template.

The genotype was confirmed via PCR and Nanopore sequencing.

**bWM200** [*amyE::erm-Paz-ftsA-ftsZ, ftsAZ::spec*] was generated by transforming bWM174 with a knockout construct created by sequential overlap extension PCR of three fragments:

1. **ftsAZ(up)**: PCR with primers oWM414 and oWM416 using PY79 genomic DNA as template.
2. **spec**: PCR with primers oDS487 and oDS488 using plasmid pWX466 as template.
3. **ftsAZ(down)**: PCR with primers oWM417 and oWM415 using PY79 genomic DNA as template.

The genotype was confirmed via PCR and Nanopore sequencing.

**bWM236** [*amyE::erm-Paz-ftsA-ftsZ(S333A), ftsAZ::spec*] was generated by transforming bWM233 with the *ftsAZ::spec* knockout construct PCR-amplified from bWM200 genomic DNA. The genotype was confirmed via PCR and Nanopore sequencing.

**bWM237** [*amyE::erm-Paz-ftsA-ftsZ(S333E), ftsAZ::spec*] was generated by transforming bWM234 with the *ftsAZ::spec* knockout construct PCR-amplified from bWM200 genomic DNA. The genotype was confirmed via PCR and Nanopore sequencing.

**bAB309** [*amyE::erm-Phyperspank-ftsA-HaloTag-Linker(15aa)-ftsZ*] was generated by A. Bisson-Filho by transforming PY79 with a Gibson assembly consisting of three fragments:

1. **amyE(up)-erm-Phyperspank-ftsA**: PCR with primers oMD191 and oAB13 using bAB98 genomic DNA as template. bAB98 [*amyE::erm-Phyperspank-ftsAZ*] is an unpublished strain similar to bWM98; the amplified region is identical to the corresponding region of bWM98.
2. **HaloTag-Linker(15aa)**: PCR with primers oJE32 and oAB14 using synthetic double-stranded DNA encoding the HaloTag protein codon-optimized for *B. subtilis* (obtained from DNA 2.0, now ATUM) as template.
3. **Linker(15aa)-ftsZ-amyE(down)**: PCR with primers oAB140 and oMD197 using bAB98 genomic DNA as template. The amplified region is identical to the corresponding region of bWM98.

The genotype was confirmed via PCR and Sanger sequencing.

**bWM8** [*amyE::erm-Phyperspank-ftsA-HaloTag-ftsZ(S329A)*] was generated by transforming PY79 with a Gibson assembly consisting of three fragments:

1. **amyE(up)-erm(5’)**: PCR with primers oMD191 and oWM113 using bWM98 genomic DNA as template.
2. **erm(3’)-Phyperspank**: PCR with primers oWM112 and oDS488 using bWM98 genomic DNA as template.
3. **ftsA-HaloTag-ftsZ(S329A)-amyE(down)**: Created by overlap extension PCR of:
   - PCR with primers oAB78 and oWM71 using bAB309 genomic DNA as template.
   - PCR with primers oWM70 and oMD197 using bWM98 genomic DNA as template.

The genotype was confirmed via PCR and Sanger sequencing.

**bWM9** [*amyE::erm-Phyperspank-ftsA-HaloTag-ftsZ(S333A)*] was generated by transforming PY79 with a Gibson assembly consisting of three fragments:

1. **amyE(up)-erm(5’)**: PCR with primers oMD191 and oWM113 using bWM98 genomic DNA as template.
2. **erm(3’)-Phyperspank**: PCR with primers oWM112 and oDS488 using bWM98 genomic DNA as template.
3. **ftsA-HaloTag-ftsZ(S333A)-amyE(down)**: Created by overlap extension PCR of:
   - PCR with primers oAB78 and oWM2 using bAB309 genomic DNA as template.
   - PCR with primers oWM1 and oMD197 using bWM98 genomic DNA as template.

The genotype was confirmed via PCR and Sanger sequencing.

**bWM10** [*amyE::erm-Phyperspank-ftsA-HaloTag-ftsZ(S329A,S333A)*] was generated by transforming PY79 with a Gibson assembly consisting of three fragments:

1. **amyE(up)-erm(5’)**: PCR with primers oMD191 and oWM113 using bWM98 genomic DNA as template.
2. **erm(3’)-Phyperspank**: PCR with primers oWM112 and oDS488 using bWM98 genomic DNA as template.
3. **ftsA-HaloTag-ftsZ(S329A,S333A)-amyE(down)**: Created by overlap extension PCR of:
   - PCR with primers oAB78 and oWM203 using bAB309 genomic DNA as template.
   - PCR with primers oWM204 and oMD197 using bWM98 genomic DNA as template.

The genotype was confirmed via PCR and Sanger sequencing.

**bWM106** [*amyE::erm-Phyperspank-ftsA-HaloTag-ftsZ, prkC::lox72*] was generated by transforming bYS542 [*prkC::lox72*] (Sun et al., 2023) with a Gibson assembly consisting of three fragments:

1. **amyE(up)-erm(5')**: PCR with primers oMD191 and oWM113 using bWM98 genomic DNA as template.
2. **erm(3')-Phyperspank**: PCR with primers oWM112 and oDS488 using bWM98 genomic DNA as template.
3. **ftsA-HaloTag-Linker(15aa)-ftsZ-amyE(down)**: PCR with primers oAB78 and oMD197 using bAB309 genomic DNA as template.

The genotype was confirmed via PCR and Sanger sequencing.

**Plasmid Construction**

**pWM1** [pET-28a::6xHis-TEV-FtsZ] was generated by synthesizing full-length *B. subtilis* FtsZ (Genscript) with an N-terminal 6xHis tag and TEV cleavage site designed for scarless cleavage, and cloning into pET-28a using NcoI and BamHI restriction sites.

**pWM2** [pET-28a::6xHis-TEV-FtsZ_Core] was created by introducing a triple stop codon into pWM1 after residue 315 using the Q5 Site-Directed Mutagenesis kit (NEB).

**pWM3** [pGEX-6P-3::GST-3C-FtsZ_Tail] was generated by synthesizing the isolated FtsZ tail (residues 316-382; Genscript) and cloning into pGEX-6P-3 using BamHI and NotI restriction sites. Restriction cloning introduced an additional LGS sequence between the PreScission protease cleavage site and the start of the FtsZ tail; this sequence was subsequently removed using the Q5 Site-Directed Mutagenesis kit, leaving only the standard PreScission GP scar.

**pWM4** [pGEX-6P-1::GST-3C-MinC_N_] was created by amplifying MinC_N_ from the *B. subtilis* genome using primers oWM612 and oWM613. The PCR product was then combined with BamHI/NotI-digested pGEX-6P-1 vector via Gibson assembly.

Point mutations for FtsZ variants (S333A, S333E, I334E, K335A/H337A) were introduced using the Q5 Site-Directed Mutagenesis kit with primers designed using the NEBaseChanger tool.

**Expression Construct Sequences**

**pWM1 insert:** 6xHis-TEV-FtsZ(WT) + NcoI/BamHI

CCATGGGCAGCAGCCATCATCATCATCATCACAGCAGCGGCGAGAATCTTTATTTTCAGATGTTGGAGTTCGAAACAAACATAGACGGCTTAGCATCAATTAAAGTAATCGGAGTAGGAGGCGGCGGTAACAACGCCGTTAACCGAATGATTGAAAATGAAGTGCAAGGAGTAGAGTATATCGCGGTAAACACGGACGCTCAAGCTCTTAACCTGTCAAAAGCAGAAGTGAAAATGCAAATCGGCGCAAAGCTGACTAGAGGATTGGGAGCAGGTGCGAATCCGGAAGTCGGGAAAAAAGCCGCTGAAGAAAGCAAAGAGCAGATTGAAGAAGCACTTAAAGGTGCTGACATGGTATTCGTGACAGCTGGTATGGGCGGCGGAACAGGAACAGGTGCCGCACCGGTTATCGCACAAATCGCGAAAGACTTAGGCGCATTAACAGTCGGCGTTGTGACAAGACCGTTTACCTTCGAAGGACGCAAAAGACAGCTTCAGGCTGCAGGCGGAATCTCGGCAATGAAAGAAGCGGTGGATACACTGATCGTGATCCCGAACGACCGTATCCTTGAAATTGTTGATAAAAACACACCGATGCTTGAAGCATTCCGCGAAGCGGATAACGTACTTCGCCAAGGGGTTCAAGGTATTTCTGACTTGATTGCTACACCTGGTCTTATCAACCTTGACTTTGCTGATGTGAAAACAATCATGTCAAACAAAGGATCTGCTTTGATGGGTATCGGTATTGCTACTGGGGAAAATCGCGCGGCAGAGGCAGCAAAAAAAGCAATTTCCAGCCCGCTTCTTGAAGCGGCCATTGACGGTGCGCAAGGCGTCCTCATGAACATCACTGGAGGAACAAACCTCAGCCTATATGAGGTTCAGGAAGCAGCAGACATTGTCGCTTCGGCGTCTGATCAAGACGTAAACATGATTTTCGGTTCTGTTATTAATGAAAATCTAAAAGATGAGATTGTGGTGACAGTGATTGCAACCGGCTTTATCGAACAAGAGAAGGACGTGACGAAGCCTCAGCGTCCAAGCTTAAATCAAAGCATCAAAACACACAATCAAAGTGTTCCGAAGCGTGAGCCAAAACGTGAGGAACCTCAGCAGCAGAACACAGTAAGCCGTCATACTTCACAGCCGGCTGATGATACGCTTGACATCCCGACATTCTTAAGAAACCGTAATAAACGCGGCTAAGGATCC

**pWM3 insert:** FtsZ_Tail(WT) + BamHI/NotI

GGATCCATCGAACAAGAGAAGGACGTGACGAAGCCTCAGCGTCCAAGCTTAAATCAAAGCATCAAAACACACAATCAAAGTGTTCCGAAGCGTGAGCCAAAACGTGAGGAACCTCAGCAGCAGAACACAGTAAGCCGTCATACTTCACAGCCGGCTGATGATACGCTTGACATCCCGACATTCTTAAGAAACCGTAATAAACGCGGCTAAGCGGCCGC

**pWM4 insert:** MinC_N_ + Gibson homology arms

GATCTGGAAGTTCTGTTCCAGGGGCCCATGAAGACCAAAAAGCAGCAATATGTAACAATAAAAGGAACAAAGAATGGACTAACATTGCATCTGGATGATGCGTGTTCTTTTGATGAGCTTCTCGATGGTCTTCAGAATATGCTGTCAATTGAACAATATACCGATGGAAAAGGCCAGAAAATCAGCGTTCATGTTAAGCTGGGAAATCGCTTTTTATATAAGGAGCAAGAGGAACAGCTAACCGAATTGATTGCGTCAAAGAAAGATTTGTTTGTTCATTCTATTGACAGTGAAGTCATTACTTAAATCGTGACTGACTGACGATCTGCCTCG

**Reference Sequences**

**Paz** [24]

GTATTTGTTTCCGGTTTCTTTTTTAATATTATATTGGCAATAAGTTTAGCTTTTCTGGGAGTCCATCTTGGTGTAGACTTGTATTTAGCAGGTATATTCGCATTTGGAGTCAGATTATTTCAGAATATAGCCGTTATCAGAAGAAATCTACTAACAAAGTGGACTCTTTCTAAAAAAAATAAAAAAAATGTGATATAAAAGAGGATATACATAGGATATAACGAATATTTTCAATAAACATAAAATGTGAAAAGCACATAAAAATATTCTGTTGTTATTTTTTGTTACACACTTGTAAAGCCACATTCATTGTATTGTTGTTCCGCAAATAATAGAATAGAAATGATCGAAATG**TGAGGAGG**TGCCATAGA

**Phyperspank** [25] **+ Optimal RBS** [26]

TAATGGATTTCCTTACGCGAAATACGGGCAGACATGGCCTGCCCGGTTATTATTATTTTTGACACCAGACCAACTGGTAATGGTAGCGACCGGCGCTCAGGATCCTAACTCACATTAATTGCGTTGCGCTCACTGCCCGCTTTCCAGTCGGGAAACCTGTCGTGCCAGCTGCATTAATGAATCGGCCAACGCGCGGGGAGAGGCGGTTTGCGTATTGGGCGCCAGGGTGGTTTTTCTTTTCACCAGTGAGACGGGCAACAGCTGATTGCCCTTCACCGCCTGGCCCTGAGAGAGTTGCAGCAAGCGGTCCACGCTGGTTTGCCCCAGCAGGCGAAAATCCTGTTTGATGGTGGTTGACGGCGGGATATAACATGAGCTGTCTTCGGTATCGTCGTATCCCACTACCGAGATATCCGCACCAACGCGCAGCCCGGACTCGGTAATGGCGCGCATTGCGCCCAGCGCCATCTGATCGTTGGCAACCAGCATCGCAGTGGGAACGATGCCCTCATTCAGCATTTGCATGGTTTGTTGAAAACCGGACATGGCACTCCAGTCGCCTTCCCGTTCCGCTATCGGCTGAATTTGATTGCGAGTGAGATATTTATGCCAGCCAGCCAGACGCAGACGCGCCGAGACAGAACTTAATGGGCCCGCTAACAGCGCGATTTGCTGGTGACCCAATGCGACCAGATGCTCCACGCCCAGTCGCGTACCGTCTTCATGGGAGAAAATAATACTGTTGATGGGTGTCTGGTCAGAGACATCAAGAAATAACGCCGGAACATTAGTGCAGGCAGCTTCCACAGCAATGGCATCCTGGTCATCCAGCGGATAGTTAATGATCAGCCCACTGACGCGTTGCGCGAGAAGATTGTGCACCGCCGCTTTACAGGCTTCGACGCCGCTTCGTTCTACCATCGACACCACCACGCTGGCACCCAGTTGATCGGCGCGAGATTTAATCGCCGCGACAATTTGCGACGGCGCGTGCAGGGCCAGACTGGAGGTGGCAACGCCAATCAGCAACGACTGTTTGCCCGCCAGTTGTTGTGCCACGCGGTTGGGAATGTAATTCAGCTCCGCCATCGCCGCTTCCACTTTTTCCCGCGTTTTCGCAGAAACGTGGCTGGCCTGGTTCACCACGCGGGAAACGGTCTGATAAGAGACACCGGCATACTCTGCGACATCGTATAACGTTACTGGTTTCATCAAAATCGTCTCCCTCCGTTTGAATATTTGATTGATCGTAACCAGATGAAGCACTCTTTCCACTATCCCTACAGTGTTATGGCTTGAACAATCACGAAACAATAATTGGTACGTACGATCTTTCAGCCGACTCAAACATCAAATCTTACAAATGTAGTCTTTGAAAGTATTACATATGTAAGATTTAAATGCAACCGTTTTTTCGGAAGGAAATGATGACCTCGTTTCCACCGAATTAGCTTCGACTCTCTAGCTTGAGGCATCAAATAAAACGAAAGGCTCAGTCGAAAGACTGGGCCTTTCGTTTTATCTGTTGTTTGTCGGTGAACGCTCTCCTGAGTAGGACAAATCCGCCGCTCTAGCTAAGCAGAAGGCCATCCTGACGGATGGCCTTTTTGCGTTTCTACAAACTCTTGTTAACTCTAGAGCTGCCTGCCGCGTTTCGGTGATGAAGATCTTCCCGATGATTAATTAATTCAGAACGCTCGGTTGCCGCCGGGCGTTTTTTATGCAGCAATGGCAAGAACGTTGCTCGAGGGTAAATGTGAGCACTCACAATTCATTTTGCAAAAGTTGTTGACTTTATCTACAAGGTGTGGCATAATGTGTGTAATTGTGAGCGGATAACAATTAAGCTT**TAAGGAGG**AACTACC

**15aa Linker** [27]

CTCGAGGGATCTGGCCAGGGACCGGGCTCAGGCCAAGGAAGCGGC

**HaloTag** [28]

ATGGCAGAAATCGGTACTGGCTTTCCATTCGACCCGCATTATGTGGAAGTCCTGGGCGAGAGAATGCATTACGTTGACGTGGGTCCGAGAGATGGAACTCCGGTCCTTTTTCTGCACGGGAATCCTACAAGCTCTTATGTTTGGCGCAATATCATCCCTCATGTAGCTCCGACGCATCGCTGTATTGCGCCGGACCTGATTGGTATGGGAAAATCTGATAAACCAGACCTGGGTTACTTTTTCGATGATCATGTGCGTTTCATGGATGCCTTCATTGAGGCATTAGGGCTTGAAGAAGTCGTCTTGGTGATTCATGATTGGGGCTCAGCTCTGGGATTTCACTGGGCTAAAAGAAATCCTGAACGCGTAAAAGGCATCGCGTTTATGGAGTTCATTCGTCCAATTCCGACTTGGGATGAATGGCCTGAGTTCGCGAGAGAAACATTTCAAGCATTTCGCACGACCGATGTAGGCCGGAAGTTAATCATCGATCAGAATGTCTTTATCGAAGGGACATTGCCGATGGGAGTCGTTCGTCCGTTAACAGAAGTCGAAATGGATCACTATAGAGAACCTTTTCTTAATCCTGTGGACAGAGAGCCGCTGTGGCGGTTTCCGAACGAACTGCCGATTGCAGGCGAGCCTGCTAACATTGTAGCGCTGGTTGAAGAGTATATGGATTGGCTTCATCAGTCTCCAGTTCCGAAGTTATTGTTTTGGGGTACGCCTGGCGTGCTTATTCCACCGGCCGAAGCGGCACGTTTGGCAAAAAGCCTGCCAAATTGCAAAGCCGTTGACATTGGCCCTGGACTTAACTTGCTTCAAGAGGATAACCCGGACTTAATCGGGAGCGAAATTGCCCGGTGGCTTTCTACCTTAGAAATCAGCGGCTAG

**Supplementary References**

1. Ravikumar V, Shi L, Krug K, Derouiche A, Jers C, Cousin C, et al. Quantitative Phosphoproteome Analysis of Bacillus subtilis Reveals Novel Substrates of the Kinase PrkC and Phosphatase PrpC. Mol Cell Proteomics. 2014;13: 1965–1978. doi:10.1074/mcp.M113.035949

2. Ravikumar V, Nalpas NC, Anselm V, Krug K, Lenuzzi M, Šestak MS, et al. In-depth analysis of Bacillus subtilis proteome identifies new ORFs and traces the evolutionary history of modified proteins. Sci Rep. 2018;8: 17246. doi:10.1038/s41598-018-35589-9

3. Birk MS, Charpentier E, Frese CK. Automated Phosphopeptide Enrichment for Gram-Positive Bacteria. J Proteome Res. 2021;20: 4886–4892. doi:10.1021/acs.jproteome.1c00364

4. Schultz C, Niebisch A, Schwaiger A, Viets U, Metzger S, Bramkamp M, et al. Genetic and biochemical analysis of the serine/threonine protein kinases PknA, PknB, PknG and PknL of *Corynebacterium glutamicum* : evidence for non‐essentiality and for phosphorylation of OdhI and FtsZ by multiple kinases. Mol Microbiol. 2009;74: 724–741. doi:10.1111/j.1365-2958.2009.06897.x

5. Maurya GK, Modi K, Banerjee M, Chaudhary R, Rajpurohit YS, Misra HS. Phosphorylation of FtsZ and FtsA by a DNA Damage-Responsive Ser/Thr Protein Kinase Affects Their Functional Interactions in *Deinococcus radiodurans*. Ellermeier CD, editor. mSphere. 2018;3: e00325-18. doi:10.1128/mSphere.00325-18

6. Thakur M, Chakraborti PK. GTPase Activity of Mycobacterial FtsZ Is Impaired Due to Its Transphosphorylation by the Eukaryotic-type Ser/Thr Kinase, PknA. J Biol Chem. 2006;281: 40107–40113. doi:10.1074/jbc.M607216200

7. Hardt P, Engels I, Rausch M, Gajdiss M, Ulm H, Sass P, et al. The cell wall precursor lipid II acts as a molecular signal for the Ser/Thr kinase PknB of Staphylococcus aureus. Int J Med Microbiol. 2017;307: 1–10. doi:10.1016/j.ijmm.2016.12.001

8. Silvestroni A, Jewell KA, Lin W-J, Connelly JE, Ivancic MM, Tao WA, et al. Identification of Serine/Threonine Kinase Substrates in the Human Pathogen Group B Streptococcus. J Proteome Res. 2009;8: 2563–2574. doi:10.1021/pr900069n

9. Giefing C, Jelencsics KE, Gelbmann D, Senn BM, Nagy E. The pneumococcal eukaryotic-type serine/threonine protein kinase StkP co-localizes with the cell division apparatus and interacts with FtsZ in vitro. Microbiology. 2010;156: 1697–1707. doi:10.1099/mic.0.036335-0

10. Manteca A, Ye J, Sánchez J, Jensen ON. Phosphoproteome Analysis of *Streptomyces* Development Reveals Extensive Protein Phosphorylation Accompanying Bacterial Differentiation. J Proteome Res. 2011;10: 5481–5492. doi:10.1021/pr200762y

11. Rioseras B, Shliaha PV, Gorshkov V, Yagüe P, López-García MT, Gonzalez-Quiñonez N, et al. Quantitative Proteome and Phosphoproteome Analyses of Streptomyces coelicolor Reveal Proteins and Phosphoproteins Modulating Differentiation and Secondary Metabolism. Mol Cell Proteomics. 2018;17: 1591–1611. doi:10.1074/mcp.RA117.000515

12. Hirakata T, Urabe H, Sugita T. Phosphoproteomic and proteomic profiling of serine/threonine protein kinase PkaE of *Streptomyces coelicolor* A3(2) and its role in secondary metabolism and morphogenesis. Biosci Biotechnol Biochem. 2019;83: 1843–1850. doi:10.1080/09168451.2019.1618698

13. Yagüe P, Willemse J, Xiao X, Zhang L, Manteca A, Van Wezel GP. FtsZ phosphorylation pleiotropically affects Z-ladder formation, antibiotic production, and morphogenesis in Streptomyces coelicolor. Antonie Van Leeuwenhoek. 2023;116: 1–19. doi:10.1007/s10482-022-01778-w

14. Garnier J, Osguthorpe DJ, Robson B. Analysis of the accuracy and implications of simple methods for predicting the secondary structure of globular proteins. J Mol Biol. 1978;120: 97–120. doi:10.1016/0022-2836(78)90297-8

15. Geourjon C, Deléage G. SOPMA: significant improvements in protein secondary structure prediction by consensus prediction from multiple alignments. Bioinformatics. 1995;11: 681–684. doi:10.1093/bioinformatics/11.6.681

16. McGuffin LJ, Bryson K, Jones DT. The PSIPRED protein structure prediction server. Bioinformatics. 2000;16: 404–405. doi:10.1093/bioinformatics/16.4.404

17. Lin K, Simossis VA, Taylor WR, Heringa J. A simple and fast secondary structure prediction method using hidden neural networks. Bioinformatics. 2005;21: 152–159. doi:10.1093/bioinformatics/bth487

18. Estaña A, Barozet A, Mouhand A, Vaisset M, Zanon C, Fauret P, et al. Predicting Secondary Structure Propensities in IDPs Using Simple Statistics from Three-Residue Fragments. J Mol Biol. 2020;432: 5447–5459. doi:10.1016/j.jmb.2020.07.026

19. Jones DT, Cozzetto D. DISOPRED3: precise disordered region predictions with annotated protein-binding activity. Bioinformatics. 2015;31: 857–863. doi:10.1093/bioinformatics/btu744

20. Abramson J, Adler J, Dunger J, Evans R, Green T, Pritzel A, et al. Accurate structure prediction of biomolecular interactions with AlphaFold 3. Nature. 2024;630: 493–500. doi:10.1038/s41586-024-07487-w

21. Youngman PJ, Perkins JB, Losick R. Genetic transposition and insertional mutagenesis in Bacillus subtilis with Streptococcus faecalis transposon Tn917. Proc Natl Acad Sci. 1983;80: 2305–2309. doi:10.1073/pnas.80.8.2305

22. Albert H, Dale EC, Lee E, Ow DW. Site‐specific integration of DNA into wild‐type and mutant *lox* sites placed in the plant genome. Plant J. 1995;7: 649–659. doi:10.1046/j.1365-313X.1995.7040649.x

23. Sun Y, Hürlimann S, Garner E. Growth rate is modulated by monitoring cell wall precursors in Bacillus subtilis. Nat Microbiol. 2023;8: 469–480. doi:10.1038/s41564-023-01329-7

24. Gonzy-Tréboul G, Karmazyn-Campelli C, Stragier P. Developmental regulation of transcription of the Bacillus subtilis ftsAZ operon. J Mol Biol. 1992;224: 967–979. doi:10.1016/0022-2836(92)90463-T

25. Van Ooij C, Losick R. Subcellular Localization of a Small Sporulation Protein in *Bacillus subtilis*. J Bacteriol. 2003;185: 1391–1398. doi:10.1128/JB.185.4.1391-1398.2003

26. Vellanoweth RL, Rabinowitz JC. The influence of ribosome‐binding‐site elements on translational efficiency in *Bacillus subtilis* and *Escherichia coli in vivo*. Mol Microbiol. 1992;6: 1105–1114. doi:10.1111/j.1365-2958.1992.tb01548.x

27. Bisson-Filho AW, Hsu Y-P, Squyres GR, Kuru E, Wu F, Jukes C, et al. Treadmilling by FtsZ filaments drives peptidoglycan synthesis and bacterial cell division. Science. 2017;355: 739–743. doi:10.1126/science.aak9973

28. Los GV, Encell LP, McDougall MG, Hartzell DD, Karassina N, Zimprich C, et al. HaloTag: A Novel Protein Labeling Technology for Cell Imaging and Protein Analysis. ACS Chem Biol. 2008;3: 373–382. doi:10.1021/cb800025k
