# Supplementary material for: FtsZ phosphorylation modulates tail-core binding to tune cell division in Bacillus subtilis
Source: PLoS One. 2025 Dec 29;20(12):e0337820. doi: 10.1371/journal.pone.0337820 (PMC12747378; doi:10.1371/journal.pone.0337820)
Supplement: Attachment — Submitted filename: 2025.10.26 Response to Reviewers.docx [file pone.0337820.s024.docx]

We thank both reviewers for their constructive comments, which have substantially strengthened our manuscript. Their suggestions prompted us to perform additional experiments that revealed the mechanistic basis for our initial observations: FtsZ’s tail directly binds its core through an interaction centered on S333, and phosphorylation disrupts this binding. In response to their feedback, we have:

1. **Performed extensive new experiments** including competitive binding assays between MinC and tail mutants (Reviewer 1), displacement experiments showing tail dominance over MinC (Reviewer 2), ITC analysis of core mutants to demonstrate direct competition rather than allostery (Reviewer 2), cell length measurements in rich media revealing context-dependent phenotypes (Reviewer 2), Western blots confirming comparable FtsZ expression levels (Reviewer 2), and NMR structural analysis of authentic phospho-S333 peptides.
2. **Significantly expanded the manuscript** with new Results sections describing tail-core binding and its structural basis (lines 244-312), expanded Discussion of regulatory mechanisms (lines 388-413), and many new supplementary figures and tables providing comprehensive supporting data.
3. **Addressed all technical concerns** including correcting nomenclature, adding line numbers, ensuring sequential figure citations, and including strain identifiers in all figure legends.

These revisions have transformed our initial phosphorylation study into a mechanistic analysis of how S333 serves as a phospho-switch that modulates tail-core binding, thereby affecting both FtsZ polymerization dynamics and regulatory protein access. We address each specific comment in detail below.

# Response to Reviewer #1

We thank Reviewer #1 for their positive assessment of our work and for acknowledging that our findings “suggest a regulatory role for FtsZ phosphorylation in cell cycle in *Bacillus subtilis*” and that “the conclusions drawn are generally well supported by the experiments and data.”

**Comment 1:** *"I'm intrigued by the model proposed in Figure 4. The authors mention that when the tail is phosphorylated, intramolecular tail-core binding is disrupted. Although the authors provide a reasonable structural explanation for the similar defects seen with both S333A and S333E mutations, I think these conclusions may be strengthened by testing the binding of the core residue mutants K335A/H337A to MinCN. One would expect that these mutants are not competed out by the CTL and would these FtsZ mutants be more sensitive to inhibition by MinCN for Z-ring inhibition."*

**Comment 2:** *"Similarly, do S333A and S333E CTL mutants fail to compete with MinCN binding."*

**Response to Comments 1 and 2:**

*Done. See Figures S12B-D and Results lines 333-342.*

We thank the reviewer for these excellent suggestions. We have performed the suggested competition assays by titrating MinC_N_ into full-length FtsZ with tail mutations (S333A, S333E, and K335A/H337A). As predicted, MinC_N_ bound all three tail mutants (Fig. S12B-D), whereas it did not bind wild-type full-length FtsZ (Fig. 4C). These results confirm that disrupting the tail-core interaction (either through mutations at S333 or at K335/H337) exposes the MinC_N_ binding site, providing strong support for our model of competitive binding between the CTL and MinC_N_. We have incorporated these results into the revised manuscript.

**Comment 3:** *"How does the GTPase activity of FL-FtsZ compare when extrinsic S333A/E CTL is added? Would it possible to obtain a CTL with Phosphoserine at 333 and compare the differences in the GTPase activity and critical concentration of polymerisation when extrinsically added to FL-FtsZ? I feel, these few in vitro experiments can really add value to the impact of the manuscript before being accepted for publication."*

**Response to Comment 3:**

*We have addressed this through new ITC experiments (Figure S12E, Results lines 339-342) and new NMR experiments (Figure 3C-F, S8-11, Tables S10-13, Results lines 244-312).*

We appreciate the reviewer’s suggestion and have performed additional ITC experiments that directly address this question. Our data demonstrate that exogenous tail cannot affect full-length FtsZ activity for the following reasons:

First, wild-type tail shows no detectable binding to full-length FtsZ (K_d_ > 2 μM; new Figure S12E) because each FtsZ molecule’s binding site is already occupied by its own intramolecular tail. This *cis* occupation prevents any *trans* interaction with exogenous tails, wild-type or otherwise.

Second, S333A and S333E tail mutants completely abolish binding even to the isolated FtsZ core (K_d_ > 2 μM; Figure 3B), despite the core lacking a competing intramolecular tail. Since these mutants cannot bind even an available site, they certainly cannot compete with the higher-affinity wild-type tail already occupying each site in full-length FtsZ.

Therefore, adding exogenous S333A/E tails would have no effect on FtsZ GTPase activity, as they cannot engage the binding site necessary to influence polymerization. Our ITC experiments provide more mechanistic insight than GTPase assays would, as they directly demonstrate the absence of the binding interaction required for any functional effect.

Regarding phosphorylated tail: While we successfully synthesized a 15aa phospho-peptide for our NMR studies, the full 67aa tail exceeds practical synthesis limits (~30aa). *In vitro* phosphorylation would require recapitulating the activation of PrkC—an RD kinase whose activation depends on its extracellular PASTA domains interacting with Lipid II in the membrane to drive dimerization and activation of the cytoplasmic kinase domain—which is not feasible for this study. Expression methods that directly incorporate phosphoserine (such as amber codon suppression) would require specialized genetic systems and typically yield insufficient protein for biochemical assays.

However, our new NMR data with a 15aa phospho-S333 peptide (*Figure 3C-F, S8-11, Tables S10-13, Results lines 244-312*) demonstrates that phosphorylation of S333 induces the same structural compaction of I334-K335 observed with S333E, validating our use of S333E as a phospho-mimetic throughout this study.

**Comment 4:** *"Also, although not essential, does depletion of any of the bundlers of FtsZ in S333A and S333E result in restoration of cell length to WT-like? If this not possible to test experimentally, some thoughts of the authors along division ring stabilisation by phosphor regulation could be included in the discussion."*

**Response to Comment 4:**

*Addressed conceptually below.*

We thank the reviewer for this insightful suggestion. In *B. subtilis*, the primary FtsZ filament bundlers are SepF and ZapA. SepF is membrane-associated and interacts with FtsZ's C-terminal peptide (CTP), while ZapA forms tetramers that interact with FtsZ’s core, though the binding interface is unknown.

Bundler deletions are known to make cells longer. For example, Δ*zapA* reduces FtsZ filament bundling, which impairs Z-ring condensation, which delays division, which makes cells longer. The reviewer raises an interesting question: could the increased FtsZ polymer abundance in S333 mutants (due to their lower critical concentration) compensate for reduced bundling activity in a double mutant, potentially restoring wild-type cell length?

While this is an intriguing hypothesis, testing it properly would be technically challenging. Both SepF and ZapA are encoded in multi-gene operons, so creating clean depletion strains without affecting other co-regulated genes is complex. Additionally, the relationship between bundler levels and cell length is likely non-linear, and bundler depletion could also have secondary effects on cell physiology that would be difficult to disentangle. Given these considerations and time constraints, this investigation extends beyond the scope of the current study.

However, we considered whether tail-core binding could affect bundling. After careful analysis, we concluded that tail phosphorylation likely does not directly modulate bundler function because: (1) FtsZ must polymerize before bundlers can act, and (2) once polymerized, the tail cannot rebind its core since the C-terminal surface is occupied by the next monomer. Therefore, bundlers interact with FtsZ filaments whose tails are already unable to bind their cores, regardless of phosphorylation state.

Any effect of S333 mutations on bundling would be indirect: enhanced polymerization provides more substrate for bundling proteins. Whether this increased polymer abundance compensates for or exacerbates bundling defects remains an interesting question for future investigation. We have revised our Discussion to acknowledge that S333 mutations alter polymerization dynamics, which could indirectly affect Z-ring organization through changes in the available bundling substrate.

# Response to Reviewer #2

We thank Reviewer #2 for their positive assessment that our report “provides a valuable insight into the specific role phosphorylation plays in regulating FtsZ in *B. subtilis*” and “is broadly influential as it provides a plausible explanation regarding the significance of FtsZ phosphorylation reported in multiple species.”

**Major Comments**

**Comment 1a:** "Fig. 1E: What is the WT used? Is it bWM200 or PY79? In the text they describe native ftsAZ promoter, is it the strain shown in S5B or S5D? If it is IPTG-inducible ftsAZ, then what concentration was used for expression?"

**Response to Comment 1a:**

*Done. Clarified throughout the manuscript.*

We apologize for the lack of clarity regarding strains. For Fig. 1E, all strains express the *ftsAZ* operon from an ectopic locus (*amyE*) under the native *ftsAZ* promoter (*Paz*) as shown in Fig. S4B, with the native *ftsAZ* operon knocked out as shown in Fig. S4C. The WT, S333A, and S333E *ftsZ* variants correspond to strains bWM200, bWM236, and bWM237, respectively. We have clarified this throughout the manuscript. The P*hyperspank* construct was a remnant from an earlier draft, and we have now removed it to avoid confusion.

**Comment 1b:** "Could the authors show a western blot to confirm all three (full-length FtsZ, S333A, S333E) are all produced at similar levels? This is important as slight variations in FtsZ level affect cell length as the authors indicate in the text; and this is the only physiologically relevant data presented in this report."

**Response to Comment 1b:**

*Done. See new Figure S4D.*

We performed Western blots to compare FtsZ protein levels across wild-type and mutant strains. FtsZ levels are comparable in all three strains, with variations of less than 10% relative to the wild-type control. Given that a two-fold increase in FtsZ protein is reported to reduce cell length by only 10% (Weart and Levin, 2003), the small variations we observe in protein levels (which are within the margin of error for quantitative Western blots) cannot account for the 5-7% decrease in cell length observed with the S333 mutants. This confirms that the cell length phenotype is due to the specific effects of the S333 mutations on FtsZ function rather than differences in expression levels. We have included these Western blot results as a new panel in Fig. S4D.

**Comment 1c:** "Indicating the strain numbers in the figure legend would be helpful for replicating these results in the future."

**Response to Comment 1c:**

*Done. Updated all figure legends.*

We have now included strain numbers in all figure legends to facilitate replication of our results.

**Comment 1d:** "Is this reproducible in another growth medium such as LB - if not, why?"

**Response to Comment 1d:**

*Done. Tested in CH rich medium (Figure 1F, Results lines 134-140, Discussion lines 402-413).*

As suggested, we tested whether the cell length phenotype is reproducible in rich media. LB medium is highly autofluorescent, making it incompatible with fluorescent imaging of stained membranes that is necessary to resolve individual cells within chains. We therefore used CH medium, another rich medium commonly used for *B. subtilis*.

Interestingly, while S333 mutants produce shorter cells in minimal media (5-7% reduction), they result in longer cells in rich media (S333A: 5% increase, S333E: 7% increase). We believe the most important observation here is that S333 mutations significantly impact cell size in both media conditions, confirming this residue’s fundamental involvement in cell size regulation.

Notably, in both conditions, the S333E mutation consistently produces a stronger effect than S333A, with S333A falling between wild-type and S333E. This gradient of phenotypic severity (where S333E consistently shows stronger effects than S333A) parallels the gradient of structural changes we observed via NMR (where WT < S333A < S333E < pS333). This further supports our use of S333E as a phospho-mimetic.

These context-dependent effects provide additional insights into S333’s role in integrating multiple regulatory inputs to FtsZ. This nutrient-dependent reversal of phenotype is consistent with previous work by Chien et al. (2012), who demonstrated that cell size in *B. subtilis* is regulated by metabolic sensors like UgtP, which inhibits FtsZ assembly specifically in nutrient-rich conditions. In minimal media, UgtP is sequestered away from FtsZ, while in rich media, UgtP actively interacts with FtsZ to delay division and increase cell size. However, the molecular interface between UgtP and FtsZ has not been experimentally determined.

To further explore the molecular basis for these context-dependent effects, we used AlphaFold Multimer to predict potential interaction sites between UgtP and FtsZ. Intriguingly, AlphaFold predicts that UgtP binds to FtsZ’s C-terminal polymerization surface — the same region where we have demonstrated FtsZ’s tail binds. This is not surprising, as polymerization surfaces are a logical regulatory target for any polymer system, and FtsZ’s C-terminal polymerization surface in particular is known to interact with two other division regulators (MinC, MciZ). This AlphaFold prediction suggests a model where the FtsZ tail and UgtP may compete for the same binding site on the FtsZ core.

In this competitive binding model:

1. In minimal media (low UgtP activity): S333 mutations disrupt tail-core binding, increasing the availability of the C-terminal polymerization surface for FtsZ-FtsZ interactions. This enhances FtsZ assembly and results in shorter cells.
2. In rich media (high UgtP activity): S333 mutations disrupt tail-core binding, potentially increasing UgtP’s access to FtsZ molecules. This might impair filament bundling or stability, resulting in longer cells.

This model suggests that S333 phosphorylation may modulate FtsZ's responsiveness to nutrient-dependent regulators like UgtP, providing a mechanism for integrating multiple cellular signals to fine-tune cell division.

**Comment 1e:** "If MinC has unlimited access to FtsZ in S333A/E mutants, then shouldn't we expect longer cells?"

**Response to Comment 1e:**

*Mechanistic explanation provided here.*

This is an excellent question that touches on the complexity of FtsZ regulation. Our data show that in S333A/E mutants, MinC_N_ indeed has increased access to FtsZ’s C-terminal polymerization surface, as demonstrated by our competitive binding experiments. Intuitively, one might expect this to result in longer cells, as MinC is a negative regulator of FtsZ polymerization. However, there are several key factors to consider:

1. **Competing effects on FtsZ polymerization:** S333 mutations have two opposing effects on FtsZ: they increase MinC accessibility (which would inhibit polymerization) but also significantly lower the critical concentration for FtsZ assembly (by up to 27%) as shown in Fig. 1C. Our cell length data suggests that in minimal media, the enhanced intrinsic polymerization properties of the mutants dominate over increased MinC accessibility.
2. **Physiological context of MinC activity:** MinC’s inhibitory function is spatially regulated. In *B. subtilis*, MinC is largely confined to cell poles through the MinCDJ-DivIVA system, with minimal activity at mid-cell where the Z-ring forms. Therefore, increased MinC_N_ binding to FtsZ may not translate directly to division inhibition at the division site.
3. **Dynamic competition *in vivo*:** While our *in vitro* experiments demonstrate that disruption of tail-core binding allows MinC_N_ to bind FtsZ, other proteins also compete with MinC for this same surface: other FtsZ cores (for polymerization), the FtsZ tail itself (as we have demonstrated), and potentially ZapA, EzrA, and UgtP (as predicted by AlphaFold Multimer). The net effect on cell division depends on which competitors predominate in a given cellular context, influenced by their relative concentrations, binding affinities, and subcellular localization. This competitive landscape helps explain why enhanced MinC binding capacity doesn’t necessarily translate to longer cells in our S333 mutants.
4. **Medium-dependent reversal of phenotype:** The fact that S333 mutations cause shorter cells in minimal media but longer cells in rich media suggests that nutrient-dependent regulators like UgtP (as we discuss in our response to Comment 1D) play a significant role in determining the final outcome. This supports our model that S333 serves as an integration point for multiple regulatory inputs, with the net effect depending on the specific cellular context.

This context-dependent phenotypic reversal provides important insight into how the same molecular perturbation (disruption of tail-core binding via S333 modifications) can lead to opposite physiological outcomes depending on the cellular environment.

**Comment 2a:** "ITC experiments: The dissociation constant between Core-CTT and Core-MinC interactions in the nanomolar range seems atypical. Most FtsZ partners are usually in the micromolar range (PMIDs: 22473211; 30636070)."

**Response to Comment 2a:**

*Data reanalyzed and contextualized below.*

We appreciate the reviewer’s concern about the submicromolar dissociation constants we reported. Upon careful re-analysis of our ITC data to address a systematic processing issue, we find that the tail binds the core with a K_d_ of ~228 nM, while MinC_N_ binds with a K_d_ of ~433 nM. We have updated our results table accordingly, and we have used the same processing strategy for all new ITC results to ensure consistency.

Many characterized FtsZ partners do indeed bind with micromolar affinity — for example, T. Maritima FtsA binds at ~50 μM (PMID 22473211) and C. glutamicum SepF binds at ~15 μM (PMID 32242019). However, these proteins bind FtsZ’s C-terminal peptide (CTP), not its globular core.

The core’s C-terminal polymerization surface operates in a different binding regime. While the binding affinities and structural interfaces of most core-binding partners have not been determined, those that have been biophysically characterized show notably higher affinities than CTP-binding proteins. MciZ binds this surface with submicromolar affinity (260 nM, PMID 30636070), and MinC_N_ has been measured as tight as 1 μM (PMIDs 10611296, 23577149, 29414773). Our measurements fall within this established range for FtsZ core interactions.

The distinct binding properties of these two interfaces likely reflect different evolutionary constraints. The CTP comprises only 10 residues that must maintain binding compatibility with multiple essential partners (FtsA, SepF, EzrA, MinC_C_/MinD). This requirement for promiscuous binding from so few residues likely constrains the CTP from optimizing high-affinity interactions with any single partner. In contrast, the core’s polymerization surface provides a larger physical area with diverse residues and secondary structural features, enabling each partner to evolve distinct, optimized contact points.

**Comment 2b:** "Although the following experiment is not needed, a better competition would be with FtsZ core + MinC, and titrating CTT to help support the claim 'Surprisingly, MinCN binding was completely abolished when titrated into full-length FtsZ (Fig. 3E, F). This is consistent with the CTT's higher affinity for the core (Kd = 66 nM vs 111 nM for MinCN).'"

**Response to Comment 2b:**

*Done. See Figure 4D and Results lines 344-359.*

We thank the reviewer for this excellent experimental suggestion. We performed the recommended displacement experiments, and they provide strong additional support for our binding model. See new Figure 4D and Results lines 344-359.

**Comment 3:** *"In the last sentence of Results section, the authors indicate 'these results demonstrate direct competition between the tail's S333 region and MinCN for the C-terminal polymerization surface.' Although plausible, unless the authors make specific mutations in the core to disrupt both MinC and CTT binding, it cannot be argued that this competition is direct. It could possibly be allosteric regulation."*

**Response to Comment 3:**

*Done. See Figure S7B-C and Results lines 230-242 for tail binding data.*

We thank the reviewer for raising this important point about distinguishing between direct competition and allosteric effects. To address this, we performed additional experiments with mutations in the FtsZ core at sites predicted to interact with the CTT and MinC_N_.

Previous structural work has established that MinC_N_ binds to FtsZ’s C-terminal polymerization surface through two specific interactions (Machado et al., 2025):

1. MinC_N_’s K15/K2 interact with FtsZ’s D289 in the H10-S9 loop.
2. MinC_N_’s K12/Y8 interact with FtsZ’s D213 in α-helix H8.

Our AlphaFold models predict that the CTT binds near both of these sites:

1. The CTT’s I334 is predicted to interact with a hydrophobic pocket (M292/I293/F294) in β-strand S9.
2. The CTT’s K335/H337 are predicted to interact with D213/D210 in α-helix H8.

Based on these structural observations and additional computational analyses (including DSSP secondary structure predictions and MutaBind2/I-Mutant stability calculations), we designed two core mutants to test whether the CTT and MinC_N_ share an overlapping binding interface.

1. A triple mutant in β-strand S9: M292H/I293W/F294K (HWK). This targets the core’s hydrophobic pocket predicted to interact with CTT I334, adjacent to the known MinC_N_ K15 binding site.
2. A double mutant in α-helix H8: D210A/D213A (AA). This directly disrupts both the predicted CTT K335/H337 interaction site and the known MinC_N_ K12 binding site.

ITC experiments with the HWK mutant revealed that these mutations completely abolished the binding of both CTT and MinC_N_ to the core. The dual loss of binding strongly supports our model that the CTT and MinC_N_ compete for the same physical site on the FtsZ core. Importantly, while the HWK mutations are adjacent to (rather than overlapping) the known MinC_N_ K15 binding site, they still eliminate MinC_N_ binding, suggesting that mutation of the MIF hydrophobic pocket in β-strand S9 alters the neighboring D289 residue or the H10-S9 loop conformation, localizing the effect to this specific region of the core.

The AA mutant produced more nuanced results that further support overlapping binding sites. CTT binding was either abolished or severely weakened (Kd ~501 nM compared to 228 nM for wild-type), confirming that the H8 helix contributes to CTT-core interaction, though it may be less critical than the MIF hydrophobic pocket. For MinC_N_ binding, the AA mutations caused a dramatic change in binding thermodynamics, switching from exothermic to endothermic. This is consistent with disruption of the salt bridge between MinC_N_ K12 and FtsZ D213, confirming that our mutations target a functional MinC_N_ binding determinant.

Taken together, these results demonstrate that mutations at two distinct locations within the C-terminal polymerization surface — the MIF hydrophobic pocket on S9 and the charged residues on H8 — disrupt both CTT and MinC_N_ binding. Combined with our competition assays showing that the CTT prevents MinC_N_ binding — both in cis (when part of full-length FtsZ) and in trans (when pre-bound as a separate molecule) — these data strongly support a model of direct spatial competition rather than allosteric regulation.

The CTT binding data with core mutants are included in the revised manuscript (Figures S7B-C); the MinC_N_ control experiments are presented here in the rebuttal and can be added to the manuscript if the reviewers deem it necessary.

**Comment 4:** *"In the Discussion: 'For filaments, our GTPase assays show S333 mutations affect catalysis, suggesting the tail still binds the core and thus may protect GDP-bound FtsZ interfaces from MinCN, though further structural studies will be needed to confirm this mechanism.' Why is this the case? If S333 mutants do not inhibit the core, higher GTPase activity is expected. The results from this publication may support the notion presented here: PMID: 32198113 (and should be cited)."*

**Response to Comment 4:**

*Interpretation revised. See updated Discussion lines 374-386.*

We thank the reviewer for this comment, which has prompted us to reconsider our interpretation. You are absolutely right that our original statement was unclear and potentially incorrect.

We wrote "suggesting the tail still binds the core” but realize this was ambiguous. We meant to say: "suggesting that **wild-type** tail **also** binds the core **even in its polymeric state**." However, upon further reflection, we recognize this interpretation was unnecessarily complex.

The enhanced GTPase activity in S333 mutants likely has a simpler explanation: increased polymerization. Since the tail binds the C-terminal polymerization surface, wild-type tails compete with other FtsZ monomers for this surface, reducing overall polymerization. S333 mutations that abolish tail-core binding eliminate this competition, leading to more polymer formation and consequently higher GTPase activity (since GTP hydrolysis occurs at polymer interfaces; more polymer = more hydrolysis).

This simpler interpretation is fully consistent with:

- Our observation that S333 mutations lower the critical concentration
- The enhanced GTPase activity of S333 mutants
- Cohan et al.'s finding that the tail acts as an auto-inhibitor

We will revise this paragraph to remove speculation about tail-polymer interactions and instead focus on how tail-core binding modulates polymerization, which in turn affects GTPase activity. We appreciate the reviewer prompting us to reconsider this interpretation.

**Comment 5:** *"'Whereas its homologs phosphorylate FtsZ in other bacteria (Supp. Table 1), such a direct link to division has not been shown in B. subtilis.' Phosphoproteomics data is experimental evidence, as such it is a direct link. Although, I agree in B. subtilis the explicit connection has not been made. As a side note, it appears that residue T323 is also phosphorylatable (PMID: 34473931)."*

**Response to Comment 5:**

*Text clarified and reference added. See Results lines 428-439.*

We appreciate the reviewer's point regarding phosphoproteomics data. We completely agree that phosphoproteomics provides direct experimental evidence of phosphorylation, and we should have been more precise in our language. What we intended to convey was that while previous studies had identified FtsZ phosphorylation sites in *B. subtilis*, the functional consequences of these modifications for cell division have not been previously characterized.

We thank the reviewer for pointing us to the additional evidence from Birk et al. (PMID 34473931) that further confirms FtsZ phosphorylation. We have incorporated this reference into our revised manuscript. Regarding T323, we could not find this site mentioned in the cited paper, which instead reports S329/S333.

However, their mass spectrometry-based approach cannot distinguish between S329 and S333 phosphorylation. Following trypsin digestion, both sites are contained within the same peptide, so phosphorylation at either position produces the same peptide mass. While Birk et al. use MaxQuant software that attempts to determine the specific phosphorylation site based on MS/MS fragmentation data, such localization is often unreliable when potential sites are so closely spaced. Indeed, examining their data reveals that both S329 and S333 are reported as phosphosites, likely reflecting this localization uncertainty. This same ambiguity also affected the two earlier Ravikumar et al. studies that we cite (PMID **24390483**, PMID **30467398**).

To definitively resolve which site is phosphorylated, we employed PhosTag gel analysis of specific point mutants. Our results unambiguously demonstrate that S333 is the phosphorylated residue under our growth conditions, as the S329A mutant retains the phosphorylated band while the S333A mutant completely eliminates it. This genetic approach provides clarity that mass spectrometry-based methods cannot achieve for closely spaced potential phosphorylation sites.

We have revised the text to more accurately state: "While phosphoproteomics studies have detected this modification in *B. subtilis* [refs], the precise site and functional consequences remained unclear. We demonstrate that PrkC phosphorylates FtsZ specifically at S333, where the tail binds the core’s polymerization surface, and we reveal the structural mechanism by which phosphorylation disrupts this interaction …".

**Minor Comment 1:** *"Line numbering would be useful for making comments."*

**Response to Minor Comment 1:**

*Done. Line numbers added throughout.*

**Minor Comment 2:** *"Supplementary figure callouts are not sequential in the text."*

**Response to Minor Comment 2:**

*Done. Sequential numbering restored.*

**Minor Comment 3:** *"prkC gene name vs PrkC protein name - standard nomenclature is not being followed. For example, in Fig. 1B it should be ΔprkC."*

**Response to Minor Comment 3:**

*Done. Nomenclature standardized throughout.*

**Minor Comment 4:** *"For the phostag gel experiments, I don't see Halo-S333E in the strain list."*

**Response to Minor Comment 4:**

*Clarification provided. S333E not needed for PhosTag analysis.*

We thank the reviewer for their careful examination of our strain list. The reviewer is correct that we did not include a Halo-FtsZ(S333E) strain for the PhosTag gel experiments. This was intentional, as PhosTag specifically binds to phosphate groups and does not interact with glutamate residues.

Each PhosTag molecule coordinates two divalent metal cations, which then interact electrostatically with phosphate groups (Kinoshita et al., 2006). This interaction is highly specific for phosphate groups and does not occur with negatively charged amino acids like glutamate or aspartate. Therefore, an S333E mutant would migrate identically to wild-type unphosphorylated FtsZ on a PhosTag gel, providing no additional information beyond what our S333A control already demonstrates.

The S333A mutant serves as an appropriate negative control, as it cannot be phosphorylated and thus shows only the lower (unphosphorylated) band in Figure 1B. This single band in the S333A lane confirms that: (1) the upper band in wild-type FtsZ represents the S333-phosphorylated form, and (2) no other phosphorylation sites are detected under our growth conditions.

We have clarified this point in the Methods section to help readers understand why only the S333A control was necessary for these experiments: “PhosTag specifically binds phosphate groups and does not interact with phosphomimetic residues (e.g., glutamate), therefore only phospho-null S-to-A mutants were used as controls alongside wild-type FtsZ.”

**Minor Comment 5:** *"NMR: Is there data for phosphomutants to show the chemical shift changes are not observed with a S333 mutant?"*

**Response to Minor Comment 5:**

*Experiment performed. Results described below.*

We repeated our NMR binding experiment with S333E mutant tail. While we do observe some chemical shift perturbations, they are substantially fewer than with wild-type tail. Given that ITC shows no detectable binding for S333E tail (Fig. 3B), these limited NMR changes likely represent weak, transient interactions rather than the stable, high-affinity binding observed with wild-type tail. This is consistent with NMR’s greater sensitivity to transient molecular encounters and confirms that S333 mutations disrupt the tail-core interaction. These data can be added to the manuscript if the reviewers deem it necessary.

**Minor Comment 6:** "Selection of H337A needs some introduction."

**Response to Minor Comment 6:**

*Done. See new Results section (lines 154-176) and new Figures 2C and S5A.*

We thank the reviewer for prompting us to clarify our selection of H337A. In the revised manuscript, we have added a new Results section (“NMR identifies core binding residues on the tail”, Results lines 154-176) that provides the experimental basis for this choice.

Briefly, we used NMR spectroscopy to partially map the tail-core binding interface. We synthesized a 15aa peptide (P328-P342) encompassing the S333 region and used ^15^N natural abundance ^1^H-^15^N HSQC and ^1^H-^1^H NOESY experiments to assign peaks to specific residues. By overlaying HSQC spectra of this peptide with our ¹⁵N-labeled full-length tail (Fig. S5, S6), we identified seven residues involved in the interaction: L330, Q332, S333, I334, K335/N338, and H337. Quantitative analysis of tail spectra at these positions revealed that addition of core induces a 32% decrease in H337 peak intensity, indicating a change in its chemical environment upon addition of core (Fig. 2C).

This experimental identification of H337 as a key binding residue, combined with AlphaFold predictions suggesting H337 may contact core residues D210/D213, led us to include H337A in our mutagenesis studies. The loss of specific binding observed with the K335A/H337A double mutant (Fig. S7A) validates the importance of these residues in the tail-core interaction.

**Minor Comment 7:** *"Could helix H10 and beta strand S9 be labeled in the figure?"*

**Response to Minor Comment 7:**

*Done. Labels added to Figure S1.*

**Minor Comment 8:** *"Could the authors discuss the mechanism and/or interaction surface used by MciZ (PMIDs: 25848052; 30636070)? This could add more value to their study if this CTL-core interaction is a general mechanism for competing with negative regulators. Also, how does interaction partners that bind to CTP affect the significance/relevance of phosphorylation (PMID: 23457247)? A brief discussion on this could also be beneficial."*

**Response to Minor Comment 8:**

*Done. See expanded Discussion (lines 388-400 for MciZ, lines 402-413 for UgtP).*

We thank the reviewer for these thoughtful suggestions about placing our findings in the broader context of FtsZ regulation. We have expanded our Discussion to explore how the tail-core interaction relates to other known FtsZ regulators and to consider the implications for CTP-binding partners. Here we provide a detailed comparison of binding mechanisms:

MciZ primarily binds the FtsZ core via beta sheet extension (PMID 25848052):

1. MciZ’s β-strand S2 interacts with FtsZ’s β-strand S9 via hydrogen bonding.
2. MciZ’s α-helix H1 interacts with FtsZ’s α-helix H10 via hydrophobic interactions.

Together, these interactions produce a binding affinity of 260 nM (PMID 30636070). Notably, MciZ binding also displaces α-helix H8.

MinC_N_ primarily binds the FtsZ core via two interactions (PMID 39793890):

1. MinC_N_’s K15/K2 interact with FtsZ’s D289 in the H10-S9 loop.
2. MinC_N_’s K12/Y8 interact with FtsZ’s D213 in α-helix H8.

Together, these produce a binding affinity that has been measured as tight as 1 μM (PMIDs 10611296, 23577149, 29414773), though K_d_ is highly sensitive to buffer conditions (both salt and pH) since contact is primarily driven by ionic interactions.

Our results suggest the FtsZ tail may bind the core via at least two interactions:

1. The tail’s I334 interacts with the core’s β-strand S9 at its MIF hydrophobic pocket.
2. The tail’s K335/H337 interact with the core’s α-helix H8 at D213/D210.

AlphaFold predicts additional hydrogen bonding contacts, but confirmation would require solving the core/tail structure via NMR. Together, these produce a binding affinity of 240 nM.

Notably, MciZ, MinC_N_, and the FtsZ tail all target overlapping regions of the C-terminal polymerization surface (S9, H10, H8), establishing this as a regulatory hotspot. The spatial proximity of the tail’s exit point (S10) to this surface suggests it is well-positioned to modulate access to this regulatory hub. While we demonstrate this directly for MinC_N_, the implications extend to other regulators.

MciZ has been reported to bind both full-length and truncated FtsZ in pull-down assays (PMID 30636070), suggesting tail-core binding might not affect MciZ activity. However, other FtsZ-binding proteins like UgtP, whose binding interface remains uncharacterized, could potentially be affected if it targets this same surface. Our observation that S333 mutations produce opposite cell length phenotypes in rich versus minimal media provides indirect support for tail-mediated regulation of nutrient-dependent factors like UgtP, but this would require structural confirmation.

Polymerization surfaces are logical regulatory targets for any polymer system, and it is easy to imagine how tail-core binding might regulate access to this surface. Beyond core interactions, tail phosphorylation may also influence CTP accessibility. When bound to the core, the tail could restrict access of CTP-binding partners like FtsA, SepF, and MinC_C_/MinD. This raises the intriguing possibility that S333 phosphorylation coordinates regulation at both ends of FtsZ, modulating core polymerization and CTP partner binding through a single modification.
